# Effects of Multicomponent Oriental Integrative Intervention on Cognitive Function, Health Status, Life Satisfaction, and Yangsaeng of Community-Dwelling Elderly

**DOI:** 10.3390/ijerph191912113

**Published:** 2022-09-24

**Authors:** Sohyune Sok, Seyoon Kim, Eunyoung Shin, Myeongshin Kim, Youngmi Cho

**Affiliations:** 1 College of Nursing Science, Kyung Hee University, Seoul 02447, Korea; 2 Department of Nursing, Graduate School, Kyung Hee University, Seoul 02447, Korea; 3 Department of Nursing, Sun Moon University, Asan-si 31460, Korea

**Keywords:** aged, multicomponent intervention, cognition, health, life satisfaction, Yangsaeng, community

## Abstract

The elderly population in South Korea is rapidly increasing. The elderly living in the community are looking for alternative and complementary methods to improve their healthy longevity and quality of life. This study aimed to examine the effects of Multicomponent Oriental Integrative Intervention on the cognitive function, health status, life satisfaction, and Yangsaeng of the Korean elderly living in the community. A quasi-experimental study design using a pretest–posttest control group was employed. Study participants were a total of 74 elderly (intervention: *n* = 37, control: *n* = 37) in Seoul, South Korea. Multicomponent Oriental Integrative Intervention was scheduled to hold two sessions a week, a total of sixteen sessions in 8 weeks, lasting 60 min per session. The measures were the general characteristics of the study participants, the Korean version of the Mini-Mental State Examination (MMSE-K), the Korean health status measure for the elderly, the Korean elderly life satisfaction scale, and the Yangsaeng measurement tool. Data were collected from March 2022 to May 2022. There were statistically significant differences in cognitive function, health status, life satisfaction, and Yangsaeng between the two groups. Multicomponent Oriental Integrative Intervention might be an effective intervention for improving the cognitive function, health status, life satisfaction, and Yangsaeng of the elderly living in the community. Health care providers need to pay attention to Multicomponent Oriental Integrative Intervention for the elderly living in the community.

## 1. Introduction

South Korea is currently an “aged society,” with 15.7% of its total population composed of people aged 65 years or older. Such population aging is expected to progress rapidly, with over 20% of the population aged 65 years or older by 2025, ushering the country into a “super-aged society” [1]. Elderly healthcare should focus not only on disease treatment but also on the identification of health risk factors, as well as the establishment of a healthy lifestyle and attitude prior to disease occurrence that can be accomplished through health-promoting actions [2].

The use of complementary and integrative therapies (CITs) is on the rise among elderly people in the world [3,4,5]. There is increasing evidence to support the use of CITs, especially mind–body therapies, diet, and nutritional supplements used for mental disorders of aging [4,6]. In CIT, all integrative practices give the belief that wellbeing is a state of balance in the spheres encompassing spiritual, physical, and mental/emotional functioning [4,7]. Wellbeing can be achieved by leading a balanced healthy lifestyle ensured by proper nutrition, exercise, sleep habits, and the ability to regulate stress response via meditation or other mind–body practices [3,4,5,7]. CIT accentuates a holistic, patient-focused approach to healthcare and wellbeing that targets the whole person rather than a single organ system [4,5]. Historically, these practices have been directed toward a harmonious balance of mind, body, and spirit [4,6,7].

The previous literature has shown that cognitive function, health status, life satisfaction, and health-promoting actions are factors of the utmost importance in elderly healthcare [5,6,8,9,10,11]. In the East, health-promoting actions are referred to as “Yangsaeng,” and the two terms can be used interchangeably [5,11]. A decline in cognitive functions while aging can lead to mild cognitive impairments or even more severe impairments that impede everyday life, such as dementia [8,9]. Such decline is the most severe problem associated with aging, causing difficulties in maintaining daily life [6]. It is both directly and indirectly related to body functions, and it has a significant impact on one’s health status [6,9]. Many elderly people are concerned about their health status, an important factor in successful aging. Health status and cognitive functions are often linked. The majority of the elderly with declined cognitive functions may also suffer from debilitating health deterioration, as damages to their cerebral cortex cause loss of motor function and balance, resulting in a significant decline in gross motor functions, such as the ability to use stairs, change directions, and walk. Life satisfaction is a subjective measure of gratification that includes, in part, an optimistic expectation of the variables in life that can affect one’s psychology [12]. South Koreans have a low level of life satisfaction, as evidenced by a life satisfaction score of 5.9, which is lower than the average OECD score of 6.5 (on a scale of 0 to 10). Furthermore, life satisfaction steadily decreases with age [10].

Traditional elderly health-promoting actions, such as Yangsaeng, are used for health and longevity maintenance [11]. Yang-saeng means to ‘nourish life’ or to ‘protect human life,’ and it is also refered to as Sub-saeng (攝生), Do-saeng (道生), Ui-saeng (衛生), and Bo-saeng (保生) [13]. Humans can achieve emotional stability and reinforce their inner values through Yangsaeng. In addition, they can maintain bodily balance through interactions with their environment [6]. Yangsaeng is an important health principle for the elderly, as it is a health-promoting activity for health maintenance and preservation, as well as successful aging.

Currently, there are about 200 domestic and foreign studies on Yangsaeng, the majority of which are focused on the Yangsaeng measurement tool or a correlation study, with inadequacy in the interventional study. In fact, there are many types of Yangsaeng methods. Each category is not distinct from the others; however, they are inextricably linked to one another and exhibit collective characteristics [11,13,14]. When used in a clinical setting, various types of Yangsaeng methods must be applied together [11,15]. So far, research on Yangsaeng programs has either evaluated the effect after using a single or a part of a Yangsaeng method or measured the changes in the Yangsaeng level after applying oriental medicine intervention, both of which are inadequate to be called a Yangsaeng program. Yangsaeng intervention includes educational content and Geon-sin methods (keep-fit methods). The educational content covers daily habits, diet, appropriate drugs, maintenance, environment, sleep, and mental attitude. Meanwhile, Geon-sin methods include qigong, meridian exercises, foot reflexology, breathing, meditation, etc. [5,6,16,17,18,19].

Mind–body therapies, such as Yoga, Tai-chi, Gi-gong, and mindfulness-based intervention, produced positive effects on mood disorders and independent physical function [4,5,6]. Within the Meridian stimulation intervention, acupressure is a holistic form of complementary medicine based on the theoretical framework for traditional Chinese medicine (TCM) [3,6,7]. Acupressure is a safe, non-invasive, cost-effective, and easily implemented technique to support the improvement of mood disorders such as depressive symptoms in dementia [3,5,6,7].

With an aging population comes an increased interest and demand for senior health care and complementary medicine, thus necessitating a paradigm shift in health-promoting actions and health maintenance systems [20]. The new paradigm of senior Yangsaeng, a health-promoting action in oriental medicine for senior health maintenance, should be promoted.

Yangsaeng, or oriental health-promoting actions that can be practiced every day, is consistent with the Western principle of health promotion [5,6,20,21]. This study was conducted to measure its effects by applying the Multicomponent Oriental Integrative Intervention developed by the authors’ research project team based on Kim’s study [21] with the Health Promotion Model of Pender [22]. The aims of this study were to examine the effects of Multicomponent Oriental Integrative Intervention on cognitive function, health status, life satisfaction, and Yangsaeng of the Korean elderly living in the community.

In light of the above, the following hypotheses were generated:

**Hypotheses** **1.** *Scores of the cognitive function, health status, life satisfaction, and Yangsaeng of the participants in the intervention group will be higher than those of the participants in the control group*.

**Hypotheses** **2.** *Multicomponent Oriental Integrative Intervention will improve the cognitive function, health status, life satisfaction, and Yangsaeng of the Korean elderly living in the community*.

### Conceptual Framework

The Health Promotion Model of Pender [22] suggests factors affecting health-promoting actions and systematizes the process by which such factors affect health-promoting actions. It is an interventional model that helps in identifying methods that can encourage changes in human actions [23]. The key principles of the health promotion model are comprised of individual characteristics and experiences, cognition and emotions regarding an action, and the results of the actions [22]. The model explains that individual characteristics and experiences affect cognition and emotions regarding actions and that all of these factors bring health-promoting actions to life [22]. Pender’s [22] model allows for the development of relevant programs based on factors affecting health-promoting actions, as well as the operational efficiency applications of such programs [23,24].

Based on previous research, this study integrated the qigong method, meridian acupressure method, and educational programs into a Multicomponent Oriental Integrative Intervention that improves cognitive and daily functional abilities. Multicomponent Oriental Integrative Intervention was used as Pender’s [22] intervention strategy for changing health-promoting actions. It was also applied to the elderly residents of the community. The cognitive function, health status, life satisfaction, and Yangsaeng (health-promoting activities) of the elderly were evaluated comprehensively.

## 2. Material and Methods

### 2.1. Study Design and Participants 

A quasi-experimental study design using a pretest–posttest control group was employed. The study participants were elderly people aged 65 years or older who are residing in Seoul, can communicate, had a cognitive function score (Mini-Mental State Examination—MMSE) of 21 or above with no cognitive impairment or subjective memory complaints, and had agreed to participate in the study. The exclusion criteria included patients who are undergoing an acute phase of disease progression, those taking medications for cognitive functions and depression, and those participating in cognition or exercise programs of another organization. Also, elderly people with a medical diagnosis of Alzheimer’s disease, mixed dementia, and dementia were excluded from this study. The participants were grouped into intervention and control groups by using convenience sampling and random allocation methods.

The G power 3.1 program was used to calculate the sample size for the study. The intervention and control groups were calculated to have 31 participants each based on the setting effect size of 0.25, a significance level of 0.05, and a power of 0.90 [25]. The final number of participants was 74 in total, with 37 subjects in the intervention group and 37 participants in the control group. The sample size of this research was deemed appropriate.

### 2.2. Multicomponent Oriental Integrative Intervention

The Multicomponent Oriental Integrative Intervention was developed by using Pender’s Health Promotion Model [22], which draws out changes in health-promoting actions by structuralizing processes that affect them. This intervention was validated by a group of experts composed of 1 professor in oriental medicine and rehabilitation, 2 oriental medicine doctors, 1 professor in nursing, 1 geriatric nurse, and 2 head nurses in the oriental medicine ward. The goal of the program was to improve the cognitive function, health status, life satisfaction, and Yangsaeng of the elderly residents of the community. The program was scheduled to hold 2 sessions a week, a total of 16 sessions in 8 weeks, lasting 60 min per session. Such a schedule was determined based on evidence from previous research [5,6,26], which shows that 8 to 10 weeks of training program results in an improvement in education for the elderly. The program was conducted in the form of group training, with a researcher demonstrating and the participants following (researcher facilitated). The program was composed of the following phase (Table 1):

### 2.3. Measures

General characteristics of the study participants included gender, age, education, marital status, living with, religion, chronic disease, drinking, smoking, exercise, and participation in dementia prevention programs. This consisted of a total of eleven items. 

Cognitive function was evaluated by using the Korean version of the Mini-Mental State Examination (MMSE-K), which was revised from the MMSE of Folstein et al. [27] by Kweon and Park [28]. MMSE-K assesses the cognitive functions of the Korean elderly in 6 categories (orientation, memory, attention, calculation, language, and time–space construction) with 12 questions. The score ranges from 0 to 30 and a higher score indicates greater cognitive ability. In the research by Kweon and Park [28], Cronbach’s α was 0.92. In this study, Cronbach’s α was 0.94.

The “Korean health status measure for the elderly,” developed by Kim et al. [29], was used as the evaluation tool in the study. This tool consists of 24 items in 3 categories: physical (15 items), emotional (5 items), and social (4 items). A 4-point Likert scale evaluation, whose score ranges from 24 to 96, was used, with a higher score indicating a healthier status of the elderly. In this study, the reliability was Cronbach’s α = 0.93.

Life satisfaction was measured by using “the elderly life satisfaction scale” standardized for the Korean elderly by Choi [30]. This tool is comprised of 20 items in 3 categories regarding satisfaction of the past (6 items), present (9 items), and future (5 items). A 3-point scale evaluation, whose score ranges from 20 to 60, was used, with a higher score indicating a greater level of life satisfaction. The tool’s reliability was Cronbach’s α = 0.90 at the time of development. In this study, the reliability was Cronbach’s α = 0.92. 

The Yangsaeng measurement tool developed by Kim [31] was used in this study. This tool is comprised of 31 items on a 5-point scale (from a score of 1 for ‘never’ to a score of 5 for ‘always’). Specific categories include ethics training (5 items), mindfulness (4 items), dietary Yangsaeng (5 items), activity and rest Yangsaeng (4 items), exercise Yangsaeng (3 items), sleep Yangsaeng (4 items), seasonal Yangsaeng (3 items), and sexual Yangsaeng (3 items). The score ranges from 31 to 155, with a higher score indicating a satisfactory level of Yangsaeng (health promotion). At the time of development [31], the overall reliability of the measurement tool was Cronbach’s α = 0.89, and Cronbach’s α coefficients for each category were equal to 0.82, 0.75, 0.73, 0.79, 0.82, 0.72, 0.77, and 0.68, respectively. In this research, the overall reliability was Cronbach’s α = 0.82, and Cronbach’s α coefficients for each category were equal to 0.87, 0.85, 0.78, 0.88, 0.84, 0.76, 0.78, and 0.76, respectively. 

### 2.4. Procedures

This research was conducted from March 2022 to May 2022. Multicomponent Oriental Integrative Intervention for the elderly of the community was a program scheduled for 2 sessions a week, a total of 16 sessions in 8 weeks, with education and intervention in each session. The researcher directly applied the intervention to the intervention group at the auditorium of the community senior center. A pre-research survey using a questionnaire regarding general characteristics, cognitive function, health status, life satisfaction, and Yangsaeng was conducted on all participants in both the intervention and control groups. Meanwhile, the post-research survey was conducted in the same manner as the pre-research survey, at the end of the program for the intervention group, and 8 weeks after the pre-research survey for the control group. Cognitive function, health status, life satisfaction, and Yangsaeng were evaluated in all subjects, both in the intervention and control groups. The evaluation was conducted by the same research assistant from the pre-research survey procedure using a blinded method so as to mask the intervention and control groups. The evaluation was conducted without the research assistant knowing who among the participants was the experimental group or the control group. Participants were applied by the blinded method who did not know if they were in the intervention group or the control group. So, a double-blinded method was used in this study. For ethical reasons, the control group received Yangsaeng-based health education and vital sign measurements after the end of the study. 

### 2.5. Statistical Analysis

Data were analyzed using the SPSS/WIN 25.0 program. The general characteristics of the study participants were analyzed by using descriptive statistics (frequency, percentage, mean, and standard deviation). The homogeneity test for, and the difference in, the general characteristics of the study participants and study variables between the intervention group and the control group were analyzed by using the chi-square test, Fisher’s exact test, and independent *t*-test. The normality of the intervention group and the control group for the dependent variable was analyzed using the Shapiro–Wilk test. The effects of the Multicomponent Oriental Integrative Intervention were verified by using the two-way repeated measures, ANOVA.

### 2.6. Ethical Considerations 

This research was conducted upon the approval of K University’s Institutional Review Committee (KHSIRB-21-581[NA]). The researcher visited the senior center in order to explain the purpose and procedure of the research to the head of the senior center. Once he received approval, the researcher distributed an information sheet to recruit participants. The researcher explained the aim and procedure of the research to the elderly who had volunteered to participate in the study and obtained their written consent. The consent form highlighted the research purpose, program methods, data collection procedure, benefits, and discomfort that may arise upon participation and compensation to be received by the participant. The consent form explained that confidentiality and anonymity were guaranteed and that participants may withdraw from the study at any time.

## 3. Results 

### 3.1. General Characteristics of the Study Participants and Homogeneity

There was a total of 74 participants in this study, with 37 participants in the intervention group and 37 participants in the control group. There was a higher number of female participants than male participants, with 27 females in the intervention group (73.0%) and 23 females in the control group (62.2%). In terms of age, participants 75 to 79 years old or above accounted for the highest percentage in both the intervention group (16 subjects, 43.2%) and the control group (13 subjects, 37.8%). In terms of education status, middle school graduates were the most common, with 16 participants in the intervention group (43.2%) and 13 participants in the control group (35.1%). Also, the majority of the participants lived only with their spouses, with 22 participants in the intervention group (59.5%) and 17 participants in the control group (45.9%). In terms of health status, the majority of the participants suffered from chronic illness, with 33 participants each in the intervention group and the control group (89.2%). The majority of the participants were current drinkers, with 31 participants in the intervention group (83.8%) and 32 participants in the control group (86.5%). In terms of exercise habits, the majority of the participants answered that they ‘sometimes’ exercise, with 30 participants in the intervention group (81.1%) and 29 participants in the control group (78.4%). In addition, the majority of the participants had never received a dementia prevention education, with 29 participants in the intervention group (78.4%) and 30 participants in the control group (81.1%) responding that they had never participated in a dementia prevention program. In this research, a test of homogeneity on general characteristics revealed that the two groups were homogenous (Table 2).

### 3.2. Homogeneity on Study Variables before the Intervention 

In this research, the test of homogeneity on the dependent variables before the intervention revealed that there were no statistically significant differences in the level scores of cognitive function, health status, life satisfaction, and Yangsaeng between the two groups. The dependent variables were homogeneous before the intervention (Table 3).

### 3.3. Effects of Multicomponent Oriental Integrative Intervention

Before the intervention, a Shapiro–Wilk test was conducted in order to validate the normality of the dependent variables. As a result, a normal distribution of the dependent variables was confirmed with a Z score of 0.94~0.96 and a p score of 0.063~0.209. In this research, the normal distribution of the dependent variables and homogeneity of the variance was confirmed, and Mauchly’s assumption of sphericity was also met.

After the intervention, the cognition level score of the intervention group was 26.81, which was slightly higher than that of the control group (26.03). The health status score of the intervention group was 79.22, which was higher than that of the control group (55.51). In addition, the life satisfaction score of the intervention group was 40.51, which was higher than that of the control group (33.95). The Yangsaeng score of the intervention group was 121.24, which was higher than that of the control group (76.51).

The analysis of the interaction effects according to group and time between the two groups showed definite statistical differences in cognition function (F = 6.18, *p =* 0.015), health status (F = 172.05, *p* < 0.001), life satisfaction (F = 4.25, *p =* 0.043), and Yangsaeng (F = 583.45, *p* < 0.001) (Table 4).

## 4. Discussion

Similar to previous study results [3,4,5,8,9,32], Multicomponent Oriental Integrative Intervention improved the cognitive function of the elderly. It is believed that the components of Multicomponent Oriental Integrative Intervention, including health education, the qigong method (paldangum), and the acupressure method, have been effective in inducing normal patterns of brain function through knowledge and information transmission, paldangum movements, and meridian acupressure. It was confirmed that the intervention has a positive effect on the cognitive function of the elderly in the community. 

Multicomponent Oriental Integrative Intervention significantly improved the health status of the elderly, thus lending support to the study results of the prior literature [3,4,6,18,19,32]. Paldangum, the main exercise of this study’s Multicomponent Oriental Integrative Intervention, is a type of qigong that is similar to the qigong used in previous studies [5,6,18,19]. It was confirmed that the intervention brought about positive improvements in the elderly’s health status. 

Multicomponent Oriental Integrative Intervention also had a positive effect on the elderly’s life satisfaction, in line with the study results of the prior literature [3,4,6,10,12,18,32]. Since life satisfaction reflects subjective feelings, it can increase as the elderly’s chronic pain or stress subsides. The study’s Multicomponent Oriental Integrative Intervention raised the elderly’s interest by playing songs that they like during the qigong (paldangum) and acupressure methods. Also, the intervention was carried out in small groups of five to six people, thus allowing the seniors to enjoy their participation through communication and dependency. The slow body movements of the qigong method (paldangum), which are similar to stretching, have been shown to relieve back and shoulder discomfort [4,5]. The acupressure method, which involves pressing the meridian points of the body with one’s fingertips, has also been shown to improve the comfort and satisfaction of the elderly [3,5,7]. 

Multicomponent Oriental Integrative Intervention significantly improved the health-promoting action of Yangsaeng in the elderly, which is consistent with the study results of the prior literature [5,6]. Yangsaeng, a health-promoting action, includes mental Yangsaeng, dietary Yangsaeng, lifestyle Yangsaeng, activity and rest Yangsaeng, sleep Yangsaeng, sexual Yangsaeng, etc. [15]. The Yangsaeng level showed improvement as a result of knowledge expansion from the education sessions, as well as the smooth circulation of blood and energy, which were achieved through body balance, breathing control, and mind stability, as derived from the qigong method (paldangum). 

Previous studies [4,5,7,32] report that mind–body therapies such as Yoga, Tai-chi, Gi-gong, mindfulness-based cognitive therapy, and mindfulness-based intervention in CIT improved depression, anxiety, cognition, and the activities of daily living. The combination of acupressure and physical activity improved depression in dementia patients compared to physical activity alone [3]. The pathomechanism might involve neuroendocrine-mediated effects of acupressure on the neural circuits implicated in mood and affect regulation [3,5,6]. Complementary and alternative therapies may ameliorate disturbances in cognition, mood, sleep, and activities of daily living [4,5,7,32]. The primary mechanisms of action include modifications in neurotransmitter synthesis, inhibition of neurotransmitter reuptake and enzyme-induced neurotransmitter breakdown, antioxidant and anti-platelet activity, enhanced blood flow, and glucose metabolism [4,5,6,32].

This Multicomponent Oriental Integrative Intervention based on Yangsaeng philosophy consists of the qigong method and acupressure method. The qigong method has the benefit of boosting one’s immune system by tuning the autonomic nervous system, allowing the body to prevent and treat diseases through balance and harmony. In addition, the qigong method is a Yangsaeng training method that disciplines the body to achieve healthy longevity without disease. It guides and develops the body’s potential by controlling and cleansing each system and its functions through training in operational movements for posture, breathing, mental and physical relaxation, and consciousness concentration. Moreover, it prevents and treats diseases, allowing for healthy longevity [33,34]. Among the qigong methods, paldangum, a traditional health method composed of eight stages, accelerates energy circulation across the body and boosts the immune system. It also strengthens the body’s inherent weaknesses by nourishing Jung (精, mind), Qi (氣, energy), and Shin (神, Spirit). Paldangum is the most fundamental method in oriental medicine Yangsaeng, and it is a highly recommended Yangsaeng method (a health-promoting method) [5,6]. Also, applying pressure to meridians and acupoints according to the meridian flow can strengthen natural curative powers in relation to energy–blood circulation control, as well as physiological and pathological changes in the internal organs [5,7,35]. 

The qigong and acupressure methods all show the oriental principle of harmony, balance, holistic safety, and comfort as a Yangsaeng philosophy [5,6,7,32,33,34,35]. Public health professionals may promote and educate clients about the Yangsaeng philosophy such as this for improving and managing clients’ health.

Based on the results of this study, public health professionals can use this Multicomponent Oriental Integrative Intervention in the community to improve the cognitive function, health status, life satisfaction, and Yangsaeng of the elderly. In the current situation, where the elderly population is rapidly increasing, it can become an effective intervention method for senior healthcare. There is a need for future research that uses physiological indicators (e.g., blood tests, vital signs, and radiology tests) as outcome variables. There is also a need for additional research in order to validate that the effects of Multicomponent Oriental Integrative Intervention used in this study are long-lasting. Concerning the procedure, the study needs to be repeated with a wider and more heterogeneous group of participants. For the healthy aging of the elderly, the development of a complementary-alternative method and research evaluating its effects must be carried out continuously. 

As the limitations of this study, the results of this study cannot be generalized to all Korean elderly since convenience sampling was used to select the elderly of the community residing at home as its study participants. Although the sample size for the study was calculated, the number of participants is not high enough. The method needs to be further tested. In addition, there was no total control over other forms of exercise that may have been engaged in by the intervention group participants during the course of the Multicomponent Oriental Integrative Intervention. Furthermore, this method may not be applicable in other countries, especially those with no tradition of Yangsaeng.

## 5. Conclusions

In conclusion, the Multicomponent Oriental Integrative Intervention used in this study improved the cognitive function, health status, life satisfaction, and Yangsaeng level of the elderly living at home. This Multicomponent Oriental Integrative Intervention may have potential beneficial results in managing dementia-related symptoms, as well as slowing disease progression. Furthermore, it will be possible to apply Multicomponent Oriental Integrative Intervention as an effective complementary alternative method for the healthy life of the elderly. 

## Figures and Tables

**Table 1:** Contents of multicomponent oriental integrative intervention.

Phase	Order (Time)	Contents
Start phase	Greeting (5 min)	Ice breaking and explanation of the process
Training phase	Health education (10 min)	Oriental medicine health education: nutrition, sleep, activity & rest, exercise, mind health, environment, safety, and longevity (one topic for each week).
	Qigong mthod (8 Paldangum: Stage 1-8 ) (20 min)	- Stage 1: Two hands hold up the heavens - Stage 2: Drawing the bow to shoot the eagle - Stage 3: Separate heaven and earth - Stage 4: Wise owl gazes backwards or look back - Stage 5: Stay the head and shake the tail - Stage 6: Two hands hold the feet to strengthen the kidneys and waist - Stage 7: Clench the fists and glare fiercely (or angrily) - Stage 8: Lifting up the heels
	Acupressure method (20 min)	Meridian acupressure around the eyes, ears, nose, cheek, and whole face.
Ending phase	Wrap up (5 min)	Summary of the lesson and farewells statements

**Table 2:** General characteristics of the study participants and homogeneity.

Characteristics	Intervention Group (*n* = 37)	Control Group (*n* = 37)	χ^2^/t (*p*)
	*n* (%)		
Gender			0.99 (0.457)
Male	10 (27.0)	14 (37.8)	
Female	27 (73.0)	23 (62.2)	
Age			1.24 (0.748)
65~69	7 (18.9)	8 (21.6)	
70~74	10 (27.0)	8 (21.6)	
75~79	16 (43.2)	14 (37.8)	
80≤	4 (10.8)	7 (18.9)	
Education			0.67 ^†^ (0.946)
None	4 (10.8)	4 (10.8)	
Elementary school	10 (27.0)	12 (32.4)	
Middle school	16 (43.2)	13 (35.1)	
High school	7 (18.9)	8 (21.6)	
Marital status			2.62 ^†^ (0.231)
single	0 (0.0)	1 (2.7)	
married	26 (70.3)	20 (54.1)	
widowed	11 (29.7)	16 (43.2)	
Living with			1.60 (0.458)
Alone	8 (21.6)	9 (24.3)	
Spouse	22 (59.5)	17 (45.9)	
With son or daughter	7 (18.9)	11 (29.7)	
Religion			0.61 ^†^ (0.961)
Protestant	21 (56.8)	22 (59.5)	
Catholicism	9 (24.3)	7 (18.9)	
Buddhist	5 (13.5)	5 (13.5)	
None	2 (5.4)	3 (8.1)	
Chronic disease (frequency)			- (1.000)
Yes	33 (89.2)	33 (89.2)	
No	4 (10.8)	4 (10.8)	
Drinking			0.107 (1.000)
Yes	31 (83.8)	32 (86.5)	
No	6 (16.2)	5 (13.5)	
Smoking			0.08 (1.000)
Yes	8 (21.6)	9 (24.3%)	
No	29 (78.4)	28 (75.7%)	
Exercise			0.37 ^†^ (1.000)
Regular	6 (16.2)	7 (18.9)	
Sometimes	30 (81.1)	29 (78.4)	
None	1 (2.7)	1 (2.7)	
Participation in dementia prevention program			0.08 (1.000)
Yes	8 (21.6)	7 (18.9)	
No	29 (78.4)	30 (81.1)	

^†^ Fisher exact test.

**Table 3:** Homogeneity on study variables before intervention.

Variables	Intervention Group (*n* = 37) Mean ± SD	Control Group (*n* = 37) Mean ± SD	t	*p*
Cognitive function	26.32 ± 1.87	26.68 ± 2.06	−0.77	0.445
Health status	54.24 ± 10.35	56.68 ± 11.89	−0.94	0.351
Life satisfaction	34.51 ± 4.79	33.16 ± 4.54	1.25	0.217
Yangsaeng	84.84 ± 7.69	87.46 ± 7.56	−1.48	0.143

**Table 4:** Effects of Multicomponent Oriental Integrative Intervention.

Variables	Group	Pre	Post	Difference	Source	F (*p*)
		Mean ± SD		Mean (SE)		
Cognitive function	Intervention (*n* = 37)	26.32 ± 1.87	26.81 ± 2.01	0.49 (0.32)	Group	0.25 (0.616)
					Time	0.13 (0.724)
	Control (*n* = 37)	26.68 ± 2.06	26.03 ± 2.40	−0.65 (0.32)	G×T	6.18 (0.015 *)
Health status	Intervention (*n* = 37)	54.24 ± 10.35	79.22 ± 3.82	24.97 (1.41)	Group	25.26 (<0.001 *)
					Time	142.81 (<0.001 *)
	Control (*n* = 37)	56.68 ± 11.89	55.51 ± 11.90	−1.16 (1.41)	G×T	172.05 (<0.001 *)
Life satisfaction	Intervention (*n* = 37)	34.51 ± 4.79	40.51 ± 11.24	6.00 (1.79)	Group	13.87 (<0.001 *)
					Time	7.18 (0.009)
	Control (*n* = 37)	33.16 ± 4.54	33.95 ± 5.68	0.78 (1.79)	G×T	4.25 (0.043 *)
Yangsaeng	Intervention (*n* = 37)	84.84 ± 7.69	121.24 ± 3.44	36.41 (1.39)	Group	509.25 (<0.001 *)
					Time	168.67 (<0.001 *)
	Control (*n* = 37)	87.46 ± 7.56	76.51 ± 2.72	−10.95 (1.39)	G×T	583.45 (<0.001 *)

* *p* < 0.05.

## Data Availability

No new data were created or analyzed in this study. Data sharing is not applicable to this article.
